# Functional Expression of Adenosine A_3_ Receptor in Yeast Utilizing a Chimera with the A_2A_R C-Terminus

**DOI:** 10.3390/ijms21124547

**Published:** 2020-06-26

**Authors:** Abhinav R. Jain, Anne S. Robinson

**Affiliations:** 1 Department of Chemical and Biomolecular Engineering, Tulane University, 6823 St Charles Ave, New Orleans, LA 70118, USA; abhinavrjain@gmail.com; 2 Department of Chemical Engineering, Carnegie Mellon University, 5000 Forbes Ave, Pittsburgh, PA 15213, USA

**Keywords:** adenosine A_3_R, GPCR trafficking, yeast, GPCR signaling

## Abstract

The adenosine A_3_ receptor (A_3_R) is the only adenosine receptor subtype to be overexpressed in inflammatory and cancer cells and therefore is considered a novel and promising therapeutic target for inflammatory diseases and cancer. Heterologous expression of A_3_R at levels to allow biophysical characterization is a major bottleneck in structure-guided drug discovery efforts. Here, we apply protein engineering using chimeric receptors to improve expression and activity in yeast. Previously we had reported improved expression and trafficking of the chimeric A_1_R variant using a similar approach. In this report, we constructed chimeric A_3_/A_2A_R comprising the N-terminus and transmembrane domains from A_3_R (residues 1–284) and the cytoplasmic C-terminus of the A_2A_R (residues 291–412). The chimeric receptor showed approximately 2-fold improved expression with a 2-fold decreased unfolded protein response when compared to wild type A_3_R. Moreover, by varying culture conditions such as initial cell density and induction temperature a further 1.7-fold increase in total receptor yields was obtained. We observed native-like coupling of the chimeric receptor to G_ai-_Gpa1 in engineered yeast strains, activating the downstream, modified MAPK pathway. This strategy of utilizing chimeric receptor variants in yeast thus provides an exciting opportunity to improve expression and activity of “difficult-to-express” receptors, expanding the opportunity for utilizing yeast in drug discovery.

## 1. Introduction

The adenosine A_3_R was the last of the four subtypes to be discovered and was the only subtype that was deorphanized after clone identification [1]. The receptor is expressed in multiple human organs including lung, kidney and brain, and interestingly, A_3_R expression is upregulated in cancers and inflammatory conditions [2,3,4]. As adenosine levels also increase in these conditions/diseases, therapeutic intervention targeting A_3_R offers promising treatment potential [5]. Until recently, because of the “two-fold nature of A_3_R signaling”, understanding whether activation of the receptor provided protective or harmful effect was a major challenge for therapeutic development [1]. Currently, clinical trials for treatment of rheumatoid arthritis, plaque psoriasis, non-alcoholic steatohepatitis and hepatocellular carcinoma via agonist targeting of the A_3_ receptors is underway [6,7,8,9,10]. Structure-guided drug discovery will further aid in understanding of the receptor and developing highly selective drugs that minimize adverse events [6,11]. 

The yeast *S. cerevisiae* is a microbial eukaryotic host uniquely positioned to produce functional GPCRs and to characterize downstream signaling. For example, functional GPCRs have been purified from *S. cerevisiae* [12,13,14] and human GPCRs can signal upon ligand binding via an engineered MAPK response pathway [15,16,17] enabling identification of novel drug candidates [18]. In addition, yeast have been utilized to study GPCR-Gα protein interactions [19,20] and the effect of receptor dimerization on signaling responses [21]. 

Receptor–receptor chimeras have been utilized to investigate the role of domains in ligand recognition, Gα protein and β-arrestin binding events, and subsequent signaling responses [22,23]. The ligand binding or downstream signaling activity of A_3_R in yeast has been tested previously, but with no measurable activity [24,25]. The adenosine A_2A_ receptor (A_2A_R) shows exceptional expression and trafficking to the plasma membrane in yeast [14,24], unlike its closely related family member A_3_R. In this report, we utilize chimeric receptor protein engineering to produce active A_3_R receptor in yeast. Previously, we have observed improved trafficking to the plasma membrane, and improved yields of native-like active receptor for A_1_R chimeric receptor variants by utilizing the A_2A_R C-terminus [26]. Similarly, by using a human/rat chimeric tachykinin 2 receptor, we observed improved functional levels of the receptor in yeast [27]. Here, we employ a similar strategy to improve the expression and obtain active receptor for A_3_R variants.

## 2. Results

### 2.1. Construction of an A_3_/A_2A_R Chimera to Aid in Receptor Expression

**Figure 1:**
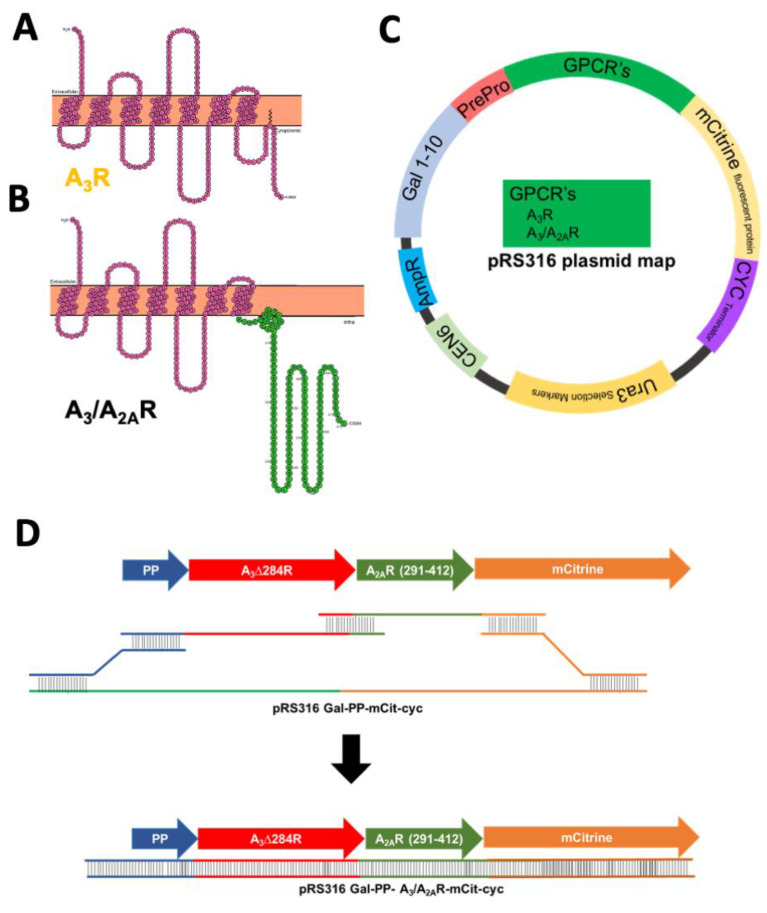
Snake plot for full-length human wild-type A_3_R (**A**) and chimeric A_3_/A_2A_R (**B**), where individual circles represent amino acids. Transmembrane helices are evident in the solid orange-colored representation of the plasma membrane. (**C**) Plasmid map for yeast expression vector pRS316-*GAL_1–10_*-PP-receptor-mCit-cyc_t_. Each receptor construct contains the *GAL_1–10_* promoter [31], an N-terminal leader sequence (PrePro [32], PP) to aid in ER trafficking, and a C-terminal mCitrine fluorescent protein (mCit) to aid in detection of receptor levels. (**D**) Schematic for homologous recombination strategy to generate chimeric A_3_/A_2A_R construct in yeast expression plasmids.

Recombinant expression of the human A_3_R in yeast has previously resulted in protein that is incapable of binding to its ligand or producing downstream activation [24,25]. On the other hand, the adenosine A_2A_R shows exceptional expression and efficient trafficking to the plasma membrane in yeast [24]. Furthermore, active A_2A_R has been purified from yeast and utilized for resolving a high-resolution crystal structure [28]. Other adenosine receptors A_1_R, A_3_R, and A_2B_R, do not show this proper trafficking in yeast, though they are membrane-integrated [24]. One major difference between A_2A_R and other adenosine receptor subtypes is the remarkably long cytoplasmic carboxy-terminus (C-terminus) of 120 amino acids. We hypothesize that this long A_2A_R C-terminus contains motifs that aid in efficient active receptor expression. Notably, for example, the C-terminus of A_2A_R contains two D/E-X-D/E motifs (located at residues 330 and 382) that would facilitate interaction with the COPII endoplasmic reticulum exit machinery [29], which is absent in A_3_R (Figure S1). Previously, a similar A_1_/A_2A_R chimera of A_1_R (1–290) and A_2A_R C-terminus (291–412) showed improved total and functional yields in yeast [26]. Note that both A_1_R and A_2A_R contain an arginine residue at 291. Although A_3_R contains a palmitoylation site at position 303, a similar site is present in A_1_R and was not included in that design, without negative impact. Therefore, to utilize these motifs and any other potential positive interactions from the A_2A_R C-terminus we constructed a chimeric A_3_/A_2A_R containing the N-terminus and transmembrane domains of A_3_R (residues 1–284) and the cytoplasmic A_2A_R C-terminal tail (291–412, Figure 1A,B). It should be noted that the chimeric variant was designed to contain helix 8 of A_2A_R.

The wild-type and chimeric receptor variants were assembled in yeast expression plasmids via homologous recombination (Figure 1C,D). To minimize the effect of plasmid copy number variation on the analysis of receptor expression, a CEN-ARS episomal centromeric plasmid (pRS316) was utilized as a template [30]. The strong, inducible galactose promoter GAL_1–10_ was used to express the receptor variants [31]. A leader peptide was added before the amino-terminus (N-terminus) of each receptor to aid in endoplasmic reticulum targeting of the GPCR [32]. Furthermore, each receptor expression construct contained a C-terminal mCitrine fluorescent protein to facilitate detection of receptor expression and trafficking. As homologous recombination with overlapping sequences was utilized to assemble the receptor variants in the plasmid, no linker was required between the leader peptide and the receptor, and the receptor and the mCitrine fluorescent protein. 

### 2.2. Improved Expression Using the Chimeric A_3_/A_2A_R 

**Figure 2:**
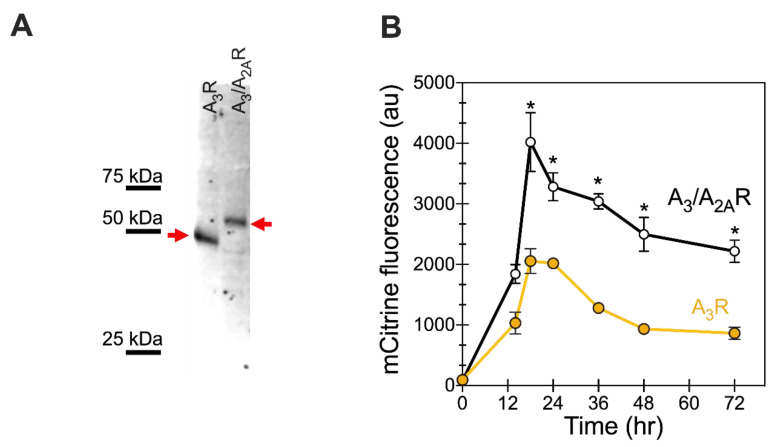
Chimeric A_3_/A_2A_R shows higher total expression levels than A_3_R in BY4741. (**A**) Representative Western blot image for cell lysates of C-terminal mCitrine-tagged receptors expressed in BY4741, 24 h post-galactose induction. One OD_600_ equivalent of whole cell lysates was loaded per well. The protein was detected using an anti-GFP antibody (see Section 4); Precision Plus Protein WesternC Standard (BioRad, Hercules, CA, USA) was used as a standard to enable molecular weight estimation. Full-length human A_3_ and chimeric A_3_/A_2A_ receptors at expected MW of 45 and 55 kDa, respectively, are identified with arrows. No cleaved mCitrine tag was observed. (**B**) Receptor expression as a function of time, following galactose induction, by monitoring whole cell mCitrine fluorescence (arbitrary units, AU) as a surrogate marker for total receptor levels. Yellow filled circles represent data for A_3_R, whereas black open circles represent A_3_/A_2A_R. Data points are the average of at least 3 biological replicates; lines are a guide for the eye. Error bars represent the standard error from the average. Data points significantly different from A_3_R are indicated with * (*p*-value < 0.05 using Student’s *t*-test).

Yeast cells were transformed with pRS316 vectors containing the A_3_ receptor variants, as described in Materials and Methods. Receptor expression was induced by growing transformed cells in galactose-containing media. No significant changes in growth were observed in recombinant cells expressing either of two receptor variants—wild-type human A_3_R and the A_3_/A_2A_R chimera. Twenty-four hours after galactose induction, cells expressing C-terminal mCitrine-tagged receptor variants were harvested for Western blot analysis (Figure 2A) to determine receptor expression. Lysates of cells expressing the receptor variants showed a prominent band at the expected MW for the full-length receptor, with no significant degradation products observed. Note that A_3_/A_2A_R has a reduced mobility (corresponding to a higher molecular weight of ~55 kDa) than the wild-type A_3_R (MW ~ 45kDa) due to the addition of the longer A_2A_R C-terminus. These data indicate that the cells are capable of producing full-length receptor variants and that the mCitrine fluorescence can serve as an appropriate surrogate for monitoring and optimizing receptor expression and tracking receptor trafficking.

Whole cell mCitrine fluorescence was monitored over time as an indirect measure of total receptor yields. The whole cell fluorescence was monitored for 72 h following galactose induction (Figure 2B). Both the wild-type and chimeric variant showed maximal expression at 18 h post galactose induction, but with different maximal fluorescence levels. The chimeric A_3_/A_2A_R (~4000 AU) showed approximately two-fold higher maximal mCitrine fluorescence levels than the wild-type receptor (~2050 AU). This higher fluorescence value suggests improved expression of the chimeric receptor as compared to the wild-type A_3_ receptor. Interestingly, after expression reached its highest value, the chimeric variant showed a slower loss of the chimeric receptor compared with that of the wild-type receptor. The total cellular productivity was determined as the area under the curve from 0 to 72 h. We observed significantly higher productivity for chimeric A_3_/A_2A_R 1.8 × 10^5^ AU (1.6–1.9 × 10^5^ AU, 95% CI) as compared with the wild-type A_3_R 8.0 × 10^4^ AU (7.3–8.8 × 10^4^ AU, 95% CI), representing an over two-fold improvement in total productivity. Taken together, these results for improved and sustained expression of the chimeric variant suggest that the presence of the A_2A_R C-terminus aids in expression of the receptor and may reduce protein turnover.

### 2.3. Decreased Unfolded Protein Response for the Chimeric A_3_/A_2A_R

**Figure 3:**
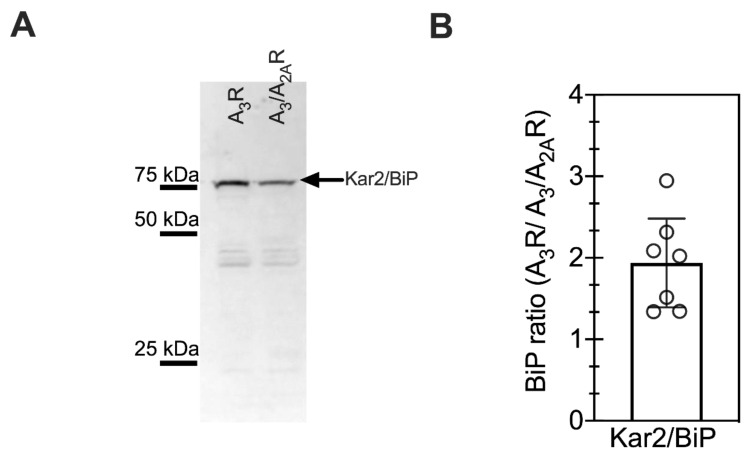
BiP/Kar2 expression was reduced in cells expressing the chimeric A_3_/A_2A_R as compared with wild-type A_3_R. (**A**) Representative Western blot of BiP/Kar2 expression levels in recombinant yeast cells. One prominent band (arrow) is observed at the expected MW for BiP/Kar2 of approximately 78 kDa. One OD_600_ equivalent of whole cell lysates were loaded per well. Precision Plus Protein WesternC Standard (BioRad) was used to enable molecular weight estimation. (**B**) Quantification of the average integrated band intensities for BiP/Kar2 levels in A_3_R-expressing cell lines from western blot images, divided by BiP levels on the same western in A_3_/A_2A_R-expressing cells to yield the fold change. Note this normalization results in no error bars for A_3_/A_2A_R data. Experiments were performed for at least four independent biological replicates; error bars represent the 95% confidence interval.

The chaperone Kar2p/BiP binds to nascent secretory and membrane proteins as they enter the endoplasmic reticulum (ER) and aids in proper folding (Figure S2). Heterologous protein expression can result in increased cell stress due to the increased protein within the ER, leading to activation of the unfolded protein response (UPR) pathway [33,34,35]. Kar2p/BiP levels are upregulated as part of UPR activation [33,34,35]. We showed previously [24] that cells expressing human A_3_R had an increased UPR as compared with A_2A_R. To investigate the effect of chimeric A_3_/A_2A_R expression on the unfolded stress response, Kar2p/BiP levels were measured in cells expressing the two A_3_ receptor variants via Western blot analysis (Figure 3A). Quantification of Western blot results from four biological replicates showed two-fold lower Kar2p/BiP levels in cells expressing the A_3_/A_2A_R variant as compared to the wild-type A_3_ receptor (Figure 3B). This result suggests that the presence of the A_2A_R C-terminus reduces the UPR stress associated with recombinant receptor expression, possibly contributing to higher receptor expression.

### 2.4. Increasing Chimeric Receptor Expression by Varying Culture Conditions

**Figure 4:**
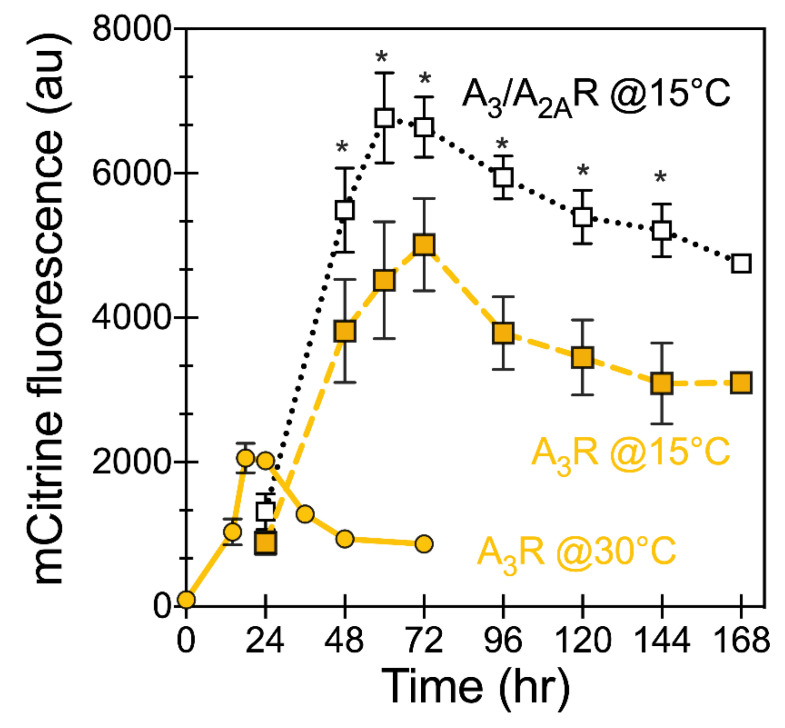
Improving receptor expression by growth at higher initial cell density (5 OD_600_/mL) and induction temperature of 15 °C. Whole cell mCitrine fluorescence was monitored by cells growing in defined media (SG-ura). Yellow-filled circles (30 °C) and squares (15 °C) denote A_3_R, whereas black open squares are A_3_/A_2A_R at 15 °C. Data points represent the average of at least 5 biological replicates; lines are a guide for the eye. Error bars represent the standard error of the mean value. Data points significantly different from A_3_R at 15 °C are indicated with * (*p*-value < 0.05 using Student’s *t*-test).

Changes in the culture conditions can lead to further improvement of receptor expression [36,37]. Previously, we have shown that varying the initial cell density at the time of galactose induction and induction temperature during protein expression had a significant impact on the total receptor yields of “difficult-to-express” receptors like A_1_R [26]. Here, we investigated these conditions for improving total receptor yields of A_3_/A_2A_R. Preliminary data showed mCitrine fluorescence levels for both of the receptor variants were approximately 25% higher when expression was induced at a ten-fold higher initial cell density of 5 OD_600_/mL. We further investigated the impact of lowering the induction temperature from 30 to 15 °C using an initial cell density of 5 OD_600_/mL on receptor expression (Figure 4, dashed lines). Expression of either of the receptors under these conditions resulted in higher mCitrine fluorescence levels, and the chimeric A_3_/A_2A_R showed around 2.4-fold improvement in fluorescent levels as compared with wild-type A_3_R grown at 15 °C at its highest point (~48 h), and approximately 3.5-fold higher than wild-type A_3_R grown at 30 °C. The total productivity (over the time course) based on total fluorescence was also higher for the chimera—5.2 × 10^5^ AU (4.6–5.9 × 10^5^ AU 95%CI) for the A_3_/A_2A_R as compared with 3.6 × 10^5^ AU (2.7–4.4 × 10^5^ AU, 95% CI) for the wild-type A_3_R. Interestingly, the A_3_/A_2A_R chimera showed a sustained expression at longer times as compared with the wild-type receptor, similar to our observation from induction at 30 °C. Taken together, by utilizing higher initial cell density and lower induction temperature we obtained significantly improved total receptor yields.

### 2.5. Improved Receptor Trafficking to the Plasma Membrane

**Figure 5:**
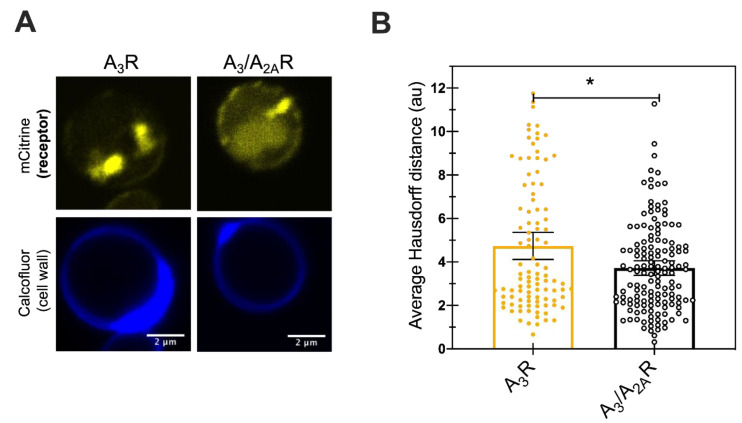
Small improvement in trafficking of chimera to the plasma membrane (**A**) Representative live-cell confocal microscopy images showing receptor localization of the mCitrine tagged receptors expressed in BY4741. Calcofluor stain was used to label the cell wall. Scale bar indicates 2 µm. (**B**) Quantification of the average Hausdorff distance for at least a hundred individual cells (points) per receptor. Bars show the mean, with error bars representing the 95% confidence interval. Student’s *t*-test was performed to determine significant difference between the two values. * represents *p*-value < 0.05.

To investigate whether the reduced UPR and improved A_3_/A_2A_R expression was a result of improved trafficking of the receptor to the plasma membrane that may be correlated with functional receptor, live cell imaging was performed. Receptor localization within the cells was determined by detecting both mCitrine (as a proxy for the C-terminal mCitrine tagged receptor location) and the fluorescent stain calcofluor, which binds to chitin in the cell wall and helps visualize the cell periphery. Figure 5A shows representative confocal micrographs for cells expressing either of the two receptors. Both showed mCitrine fluorescence predominantly inside the cells with some expression localized near the cell periphery. We quantified the results using an in-house tool that quantifies the average Hausdorff distance for individual cells [38]. The average Hausdorff distance between the pixels of mCitrine and Calcofluor stain for all mCitrine pixels was calculated for more than 100 individual cells (Figure 5B). A small number for the Hausdorff distance indicates a minimal distance between the protein and the cell periphery, indicating efficient plasma membrane trafficking of the receptor. Here, we observed Hausdorff distances of 4.7 (4.1–5.4 AU, 95%CI) for A_3_R and of 3.7 AU (3.4–4.0 AU, 95% CI) for A_3_/A_2A_R. The significantly lower Hausdorff distance for chimeric receptor variant suggests improved trafficking of the receptor to the plasma membrane. Previous studies from our laboratory had reported a Hausdorff distance of less than two arbitrary units for membrane-associated, well-trafficked receptors like A_2A_R and the yeast mating receptor (Ste_2_R) [26]. Taken together, this result suggests that the addition of the A_2A_R C-terminus improved receptor trafficking, but that there is still room for further improvement in the plasma membrane trafficking of the receptors. 

### 2.6. Chimeric A_3_/A_2A_ Receptor Was Capable of Downstream Signaling Activity in Yeast

To test whether the receptor chimeras were capable of producing native-like downstream signaling, engineered yeast strains were utilized. Yeast contains a native GPCR-mediated MAPK pheromone response pathway that has been modified to study the downstream signaling behavior of human receptors [17,39]. To obtain the proper interaction of mammalian GPCRs with the yeast Gα protein (Gpa1p), these cells express a hybrid yeast/human Gα protein. These hybrid Gα proteins contain five amino acid residues from the relevant human Gα protein that enables them to couple with a human GPCR [17,39]. Further modification was performed on these strains to obtain an easily measurable fluorescence-based response as previously described [40]. These modified strains express an mCherry fluorescent protein under control of the Fus1 promoter, which is upregulated upon receptor activation by ligand binding. Therefore, an increase in mCherry fluorescence serves as an downstream reporter of ligand-mediated receptor activation. 

**Figure 6:**
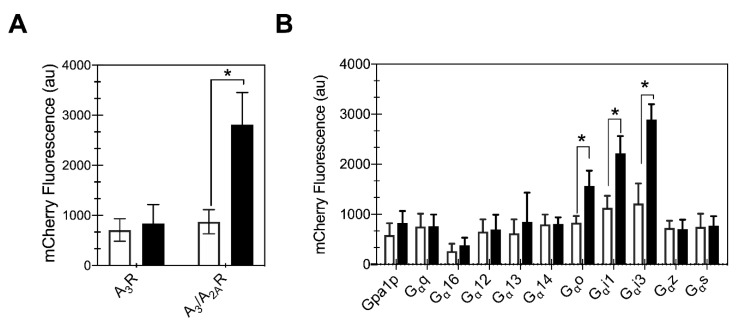
Chimeric receptor signals downstream in yeast upon ligand binding. (**A**) Chimeric A_3_/A_2A_R receptor signaling in the presence of agonist (100 μM NECA, filled bars) compared with control (DMSO, open bars) is significant, whereas wild-type A_3_R shows no detectable signaling. Both signals were measured in engineered yeast (CY13393) expressing the Gpa1p-human Gαi1 chimera and the indicated receptor. (**B**) A_3_/A_2A_R chimera is activated with native-like coupling to hybrid G_α_i/o proteins. Results obtained for engineered yeast strains with human/yeast G protein chimeras described previously [17]. The average of three independent transformants performed in duplicates is shown; error bars represent the 95% CI. * represents *p*-value < 0.05 using Student’s *t*-test.

Activation of A_3_R in mammalian cells results in a decrease in cyclic adenosine monophosphate (cAMP) levels via its interaction with inhibitory Gαi/o proteins. Consequently, a strain expressing the yeast/human Gαi1 protein (CY13393) was utilized to study the downstream signaling response of the receptors. After 24 h of galactose induction, cells expressing either of the two receptors were stimulated by addition of 100 μM 5′-*N*-ethylcarboxamidoadenosine (NECA), a high affinity non-selective adenosine receptor agonist, and whole cell mCherry fluorescence was measured (Figure 6A). As a control to determine basal activation, cells were exposed to an equivalent volume of DMSO. The A_3_/A_2A_R chimera showed a significant increase in mCherry fluorescence in agonist-treated cells compared to the DMSO control (*p* < 0.01, Student’s *t*-test), while wild-type A_3_R showed no increase in mCherry fluorescence relative to the DMSO control. This result for wild-type receptor is consistent with a previous study that reported that A_3_R was incapable of producing downstream signaling in yeast [25]. The A_3_/A_2A_R chimera activation of downstream signaling suggests that the presence of A_2A_R C-terminus aids trafficking of folded receptor that is competent to bind to the ligand and produce native-like downstream signaling.

### 2.7. Chimeric Receptor Shows Preferential Coupling with the Inhibitory Gα Protein Family 

To determine whether the chimeric receptor signaling in yeast reflects the G protein binding preferences of the native A_3_ receptor in mammalian cells, we tested the A_3_/A_2A_R chimera activity in a set of 11 yeast strains representing the GPCR-Gα interaction landscape (Table 1). The A_3_/A_2A_R chimera showed a ligand-mediated mCherry fluorescence in strains expressing hybrid Gαo, Gαi1 and Gαi3 proteins, i.e., the inhibitory Gα protein subtypes (Figure 6B). Note that the ligand-mediated Gαi1-related response for MMY23 is slightly lower than that reported for the CY13393 (Figure 6A,B), as this set of strains shows overall lower activation, but enables effective comparison of different Gα proteins. We have previously described the specificity of A_2A_R coupling in yeast to Gαs protein, and the promiscuous Gαz, which is consistent with previous reports from Knight et al. [18]. Interestingly, we did not observe increased mCherry fluorescence in cells expressing hybrid Gαs protein, as might be expected if the C-terminus of A_2A_R C-terminus affected the coupling specificity of the chimeric receptor. Taken together, we find that the chimeric A_3_/A_2A_R has native-like downstream coupling preferences for the Gα proteins.

## 3. Discussion

Obtaining high levels of A_3_R expression has been a major bottleneck for biophysical characterization and high-resolution crystallography. Previously, we utilized chimeric protein engineering of A_1_R in yeast to improve the secretory trafficking and obtained higher active receptor yields. Here, a similar strategy was utilized to improve the expression of surface localized and functional A_3_R. We observed that the A_3_/A_2A_R chimera had improved total receptor yields (approximately 2-fold higher) with reduced UPR stress as compared with wild-type A_3_R expression. The A_3_/A_2A_R chimera showed sustained expression, similar to our previous studies of A_1_/A_2A_R, suggesting the A_2A_R C-terminus may aid in reducing protein turnover [26], by an as yet unknown mechanism. The A_3_/A_2A_R chimera showed improved trafficking to the plasma membrane as compared with the wild-type A_3_ receptor. Surprisingly, both the A_3_/A_2A_R chimera and wild-type A_3_R showed inefficient receptor trafficking to the cell surface, as most of the receptor appeared to be localized inside the cells compared to the well trafficked A_2A_R described previously [24]. This was unexpected as our previous studies of A_1_/A_2A_R and A_2B_/A_2A_R showed improved receptor localization at the plasma membrane [26,41]. 

Further improvement in total receptor yields was achieved by varying culture conditions such as initial cell density and induction temperature. Receptor expression via the galactose promoter depends on the galactose level in the media. As the cells utilize galactose as a carbon and energy source, galactose is consumed during growth and its level drops over the culture time-course. Therefore, starting with higher initial cell density can result in higher protein yields [26]. Here, we observed a similar increase in total receptor yields when starting at a higher cell density (5 OD_600_/mL as compared to 0.5 OD_600_/mL). Moreover, lowering the induction temperature to 15 °C resulted in a further improvement of total yields, which is consistent with previous reports where similar improvements were observed for membrane proteins including GPCRs [36,42]. Overall, these changes in culture conditions combined with protein engineering resulted in a greater than four-fold higher receptor yield compared with wild-type A_3_R.

Downstream signaling of adenosine receptors A_1_R, A_2A_R and A_2B_R in yeast has been studied extensively [18,19,43,44,45]. To date, the downstream signaling of A_3_R has never been observed in yeast, which has been attributed primarily to inactivity of the recombinantly expressed receptor [24,25]. To the best of our knowledge, this is the first study to report active A_3_R, as observed by downstream signaling activation in the engineered yeast, which provides an exciting opportunity to screen for novel ligands targeting the A_3_R in the microbial yeast system.

## 4. Materials and Methods 

### 4.1. Cell and Culture Conditions 

*Saccharomyces cerevisiae* strains used in this study are summarized in Table 1. BY4741 (*MATa his3*Δ*1 leu2*Δ*0 met15*Δ*0 ura3*Δ*0*) was used for protein expression and trafficking experiments. Engineered yeast strains were obtained from Simon Dowell (Glaxo Smith Kline, Stevenage, UK) and the Broach laboratory (Pennsylvania State University) and were further modified to study receptor-mediated downstream signaling [40]. The parental yeast strains were grown in YPD media (2% bacto peptone, 2% glucose, 1% yeast extract). Yeast cells were transformed using lithium chloride [43]. After plasmid transformation, cells were grown in synthetic media (2% dextrose (SD) or galactose (SG), 0.67% yeast nitrogen base, 4.2 g/L citric acid and 14.7 g/L sodium citrate at pH 5.4) supplemented with amino acids and essential nutrients as per Burke et al. [46]. Uracil was omitted from synthetic media (SD-ura or SG-ura) to select for plasmid-containing cells. Individual colonies were selected and grown in culture tubes for expression studies and in 48-well plates (Cat # 353047, Corning Inc., Corning, NY, USA) for MAPK response experiments. Cells were cultured at 30 or 15 °C, as indicated, at 275 rpm. Cell growth was monitored by measuring optical density at 600 nm (OD_600_) using a Nanodrop 2000 (ThermoFisher Scientific, Waltham, MA, USA). *E. coli* strain DH5α was used for amplifying yeast expression plasmids. *E. coli* were grown in Luria–Bertani media supplemented with 100 μg/mL ampicillin at 37 °C at 250 rpm. 

### 4.2. Plasmid Construction

The A_3_R receptor variants were subcloned into yeast expression plasmids using homologous recombination in yeast [47]. A list of primers utilized for this cloning are described in Table 2. The CEN-ARS plasmid (pRS316) [30] containing a galactose (pGAL_1–10_) promoter, a N-terminal pre-pro leader sequence (PP) [32], receptor, and C-terminal mCitrine fluorescent protein and the CYC1 terminator (CYC1_t_) was utilized to express receptor variants for this study and its construction was previously described [40]. The pre-pro leader sequence aids in targeting the receptor to the plasma membrane via the secretory pathway [32]. The PCR-amplified receptor fragments and EagI linearized plasmid were combined at 4:1 molar ratios and transformed into yeast via the lithium chloride transformation protocol [48]. Transformants verified by colony PCR were miniprepped and transformed into *E. coli* using a standard heat shock protocol. All plasmids were sequenced to confirm the correct gene sequence (Genewiz, Plainfield, NJ, USA). 

### 4.3. Whole Cell Fluorescence Assay

Receptor expression was determined by measuring the fluorescence intensity of mCitrine-tagged receptor. The mCitrine fluorescent intensity was collected at excitation and emission wavelengths of 510 and 540 nm, respectively using a BioTek Synergy H1 microplate reader (Winooski, VT, USA). For mCitrine fluorescence measurements, liquid cultures of cells expressing mCitrine-tagged receptors were removed and transferred to a 96-well plate (100 μL/well) at varying time points. Experiments were performed in triplicate for three independent biological replicates. Mean and standard deviation were reported.

### 4.4. Western Blotting

Western blotting was performed as previously described [26]. Briefly, cell pellets (10 OD_600_) were resuspended in 250 μL lysis buffer (10% glycerol, 50 mM sodium phosphate monobasic and 300 mM sodium chloride at pH 8) supplemented with cOmplete EDTA-free protease inhibitor cocktails (Roche, Indianapolis, IN, USA). An equal volume (250 μL) of 0.5 mm zirconia/silica beads (BioSpec, Bartlesville, OK, USA) was added to the cells and lysed using a BeadBug homogenizer (Benchmark Scientific, Edison, NJ, USA). Cells were lysed for 4 cycles of 30 s with 1-minute rest on ice. Three parts cell lysate was mixed with one part 4X laemmli sample buffer (Biorad, Hercules, CA, USA) supplemented with β-mercaptoethanol. One OD_600_ equivalent of cell lysate was loaded per well. Rabbit polyclonal to Kar2p/BiP was produced and purified from rabbit serum, as per previously published protocol [49]. The mCitrine tagged receptors and Kar2p/BiP protein were detected using primary rabbit anti-GFP antibody (Abcam #ab6556, Cambridge, MA, USA) and rabbit polyclonal anti-BiP antibody, respectively. Primary antibody was added at 1:3000 dilution in 5% milk dissolved in Tris-buffered saline with Tween20 (20 mM Tris, 150 mM NaCl and 0.1% Tween 20, pH 7.4; TBST) followed by Alexa568 donkey anti-rabbit secondary antibody (Invitrogen, Carlsbad, CA, USA) at 1:3000 dilution in TBST buffer. Blots were imaged using a BioSpectrum imager (UVP, Upland, CA, USA). For quantifying BiP expression levels, experiments were performed in duplicates for three biological replicates. Rectangular selection tool in Image J was used to calculate integrated fluorescent signal intensities for the Kar2p/BiP band on the gels. 

### 4.5. Confocal Microscope

Live cells imaging was performed to determine receptor trafficking to the plasma membrane. The C-terminal mCitrine tagged receptors were imaged 24 h after galactose induction using a Nikon A1 laser-scanning confocal microscope. Calcoflour White M2R (Cat # F3543, Sigma Aldrich, St Louis, MO, USA), a stain that binds to chitin in the yeast cell wall was used to label the cell periphery. Excitation and emission settings of 405 nm and 450/50 nm and 513 nm and 535/15 nm were utilized to image Calcofluor stain and mCitrine fluorescent protein, respectively. Two to four images were collected for each receptor variant from four different biological replicates. Individual cells were cropped and analyzed using an in-house ImageJ plugin. The plugin calculates the average Hausdorff distance between mCitrine and Calcofluor pixels for each mCitrine pixel as previously described [26,38]. The analysis was performed for at least 100 individual cells. 

### 4.6. Pheromone Response Signaling

The downstream signaling activity of receptors was determined in engineered yeast strains as previously described [50]. Upon ligand-mediated receptor activation, the cells produce mCherry fluorescent protein via a modified MAPK response pathway. Single colonies were picked from freshly made transformants and grown overnight in 400 µL or 1mL SD-ura media in 48-well or 24-well plates (Falcon 353047 and 353078, Corning, NY, USA), respectively, at 30 °C and 275 rpm. Receptor expression was induced by transferring twelve µL of overnight culture into 400 µL SG-ura media supplemented with 0.125% glucose. This small glucose addition aids in cell growth of the engineered yeast strains without repressing the galactose promoter, as previously described [31,51]. After 24 h of galactose induction, ligand was added to activate receptor signaling. In each well of a 48-well plate, 380 µL of fresh SG-ura media, 12 µL of overnight culture, and 8 µL of 5 mM 5′-*N*-ethylcarboxamidoadenosine (NECA, solubilized in dimethyl sulfoxide; Tocris, Minneapolis, MN, USA) or dimethyl sulfoxide (DMSO, added as a control) was added and incubated at 30 °C and 275 rpm. The resulting concentration of NECA in the assay was 100 µM, which is well above the K_D_, but consistent with other yeast studies of adenosine receptor signaling [39]. After 24 h of incubation with ligand, the resulting liquid culture was transferred to three replicate wells (100 µL per well) of a 96-well plate (Costar 3915, Corning, NY, USA). Fluorescence intensity was measured using the BioTek Synergy H1 microplate reader (Winooski, VT, USA) maintained at 30 °C. Excitation and emission settings of 580 nm and 615 nm were used to determine mCherry fluorescence. Experiments were performed at least in biological duplicates from three independent transformants, for a total of six independent colonies.

## Figures and Tables

**Table 1:** List of yeast strains used in this study [15,17].

Yeast Strain	G Protein	Last 5 Amino Acids at C-Terminus	Equivalent Human G_α_
MMY12, BY4741	Gpa1	KIGII^COOH^	GPA1 (yeast)
MMY14, CY13397	Gpa1-Gαq(5)	EYNLV^COOH^	GNAQ, GNA11
MMY16, CY13395	Gpa1-Gα16(5)	EINLL^COOH^	GNA15, GNA16
MMY19	Gpa1-Gα12(5)	DIMLQ^COOH^	GNA12
MMY20	Gpa1-Gα13(5)	QLMLQ^COOH^	GNA13
MMY21	Gpa1-Gα14(5)	EFNLV^COOH^	GNA14
MMY22	Gpa1-Gαo(5)	GCGLY^COOH^	GNAO
MMY23, CY13393	Gpa1-Gαi1(5)	DCGLF^COOH^	GNAI1, GNAI2, GNAT1, GNAT2, GNAT3
MMY24	Gpa1-Gαi3(5)	ECGLY^COOH^	GNAI3
MMY25	Gpa1-Gαz(5)	YIGLC^COOH^	GNAZ
MMY28, CY13399	Gpa1-Gαs(5)	QYELL^COOH^	GNAS, GNAL

**Table 2:** List of primers used in this study.

Primer Name	Sequence	Receptor Variants
PP_A3_F	CGGTTCCGCTGCAGAAGGCTCTTTGGACAAGAGAGAAGCTATGCCCAACAACAGCACTGC	A_3_R and A_3_/A_2A_R
A3_mCit_R	TTGGGACAACACCAGTGAATAATTCTTCACCTTTAGACATCTCAGAATTCTTCTCAATGC	A_3_R
A3_A2A_F	CCATGATGAACCCTATCGTCTATGCCTATCGTATCCGCGAGTTCCGCCAGACCTTCCGCA	A_3_/A_2A_R
A3_A2A_R	TGCGGAAGGTCTGGCGGAACTCGCGGATACGATAGGCATAGACGATAGGGTTCATCATGG	A_3_/A_2A_R
A2A_mCit_R	TTGGGACAACACCAGTGAATAATTCTTCACCTTTAGACATGGACACTCCTGCTCCATCCT	A_3_/A_2A_R

## Supplementary Materials

The following are available online at https://www.mdpi.com/1422-0067/21/12/4547/s1.

## Abbreviations

A_3_/A_2A_R	Chimeric receptor comprised of N-terminus and transmembrane domains from A_3_R (residues 1–284) and the cytoplasmic C-terminus of the A_2A_R (residues 291–412)
A_3_R	Adenosine A_3_ receptor
DMSO	Dimethyl sulfoxide
NECA	5′-*N*-ethylcarboxamidoadenosine
PP	Pre-pro
