# Supplementary material for: Functional Expression of Adenosine A3 Receptor in Yeast Utilizing a Chimera with the A2AR C-Terminus
Source: Int J Mol Sci. 2020 Jun 26;21(12):4547. doi: 10.3390/ijms21124547 (PMC7352405; doi:10.3390/ijms21124547)
Supplement: Supplementary file 1 [file ijms-21-04547-s001.pdf]

**Supplement to "Functional expression of adenosine $A_3$ receptor in yeast utilizing a chimera with the $A_{2A}$R C-terminus"**

Abhinav R. Jain and Anne S. Robinson

```
CLUSTAL O(1.2.4) multiple sequence alignment

SP|P0DMS8|AA3R_HUMAN  MPNNSTALSLANVTYITMEIFIGLCAIVGNVLVICVVKLNPSLQTTTFYFIVSLALADIA 60
SP|P29274|AA2AR_HUMAN ------MPIMGSSVYITVELAIAVLAILGNVLVCWAVWLNSNLQNVTNYFVVSLAAADIA 54
                               :.. .***:*: *.: **:*****  .* ** .**..* **:**** ****

SP|P0DMS8|AA3R_HUMAN  VGVLVMPLAIVVSLGITIHFYSCLFMTCLLLIFTHASIMSLLAIAVDRYLRVKLTVRYKR 120
SP|P29274|AA2AR_HUMAN VGVLAIPFAITISTGFCAACHGCLFIACFVLVLTQSSIFSLLAIAIDRYIAIRIPLRYNG 114
                      ****.:*:**.:* *:    :.***::*::*::*::**:******:***: ::: :**:

SP|P0DMS8|AA3R_HUMAN  VTTHRRIWLALGLCWLVSFLVGLTPMFGWNMKLTSEYHR------NVTFLSCQFVSVMRM 174
SP|P29274|AA2AR_HUMAN LVTGTRAKGIIAICWVLSFAIGLTPMLGWNNCGQPKEGKNHSQGCGEGQVACLFEDVVPM 174
                      :.*  *    :.:**::** :*****:***     :  :      .   ::* * .*: *

SP|P0DMS8|AA3R_HUMAN  DYMVYFSFLTWIFIPLVVMCAIYLDIFYIIRNKLSLNLSN---SKETGAFYGREFKTAKS 231
SP|P29274|AA2AR_HUMAN NYMVYFNFFACVLVPLLLMLGVYLRIFLAARRQLKQMESQPLPGERARSTLQKEVHAAKS 234
                      :*****.*:: :::**::* .:** **   *.:*.   *:   .:.: :   :*.::***

SP|P0DMS8|AA3R_HUMAN  LFLVLFLFALSWLPLSIINCIIYFNGE---VPQLVLYMGILLSHANSMMNPIVYAYKIKK 288
SP|P29274|AA2AR_HUMAN LAIIVGLFALCWLPLHIINCFTFFCPDCSHAPLWLMYLAIVLSHTNSVVNPFIYAYRIRE 294
                      * ::: ****.**** ****: :*  :   .*  ::*:.*:***:**::**::***:*::

SP|P0DMS8|AA3R_HUMAN  FKETYLLILKACVVCHPSDSL-----------------DTSIEKNSE------------- 318
SP|P29274|AA2AR_HUMAN FRQTFRKIIRSHVLRQQEPFKAAGTSARVLAAHGSDGEQVSLRLNGHPPGVWANGSAPHP  354
                      *::*:  *::: *: : .                    :.*:. *..

SP|P0DMS8|AA3R_HUMAN  ----------------------------------------------------------
SP|P29274|AA2AR_HUMAN ERRPNGYALGLVSGGSAQESQGNTGLPDVELLSHELKGVCPEPPGLDDPLAQDGAGVS 412
```

Figure S1 (A) Pairwise sequence alignment of $A_{2A}$R (P29274) and $A_3$R(P0DMS8) generated using the UNIPROT alignment tool. Transmembrane helices in $A_{2A}$R and putative transmembrane helices in $A_3$R are underlined, and the amino acids in bold. Identical amino acids denoted by *; conservative changes by period or colon. The palmitoylation site in $A_3$R (Cys 303) is double underlined, and colored red. The two D/E-X-D/E motifs in $A_{2A}$R (located at residues 330 and 382) are boxed and highlighted in yellow.

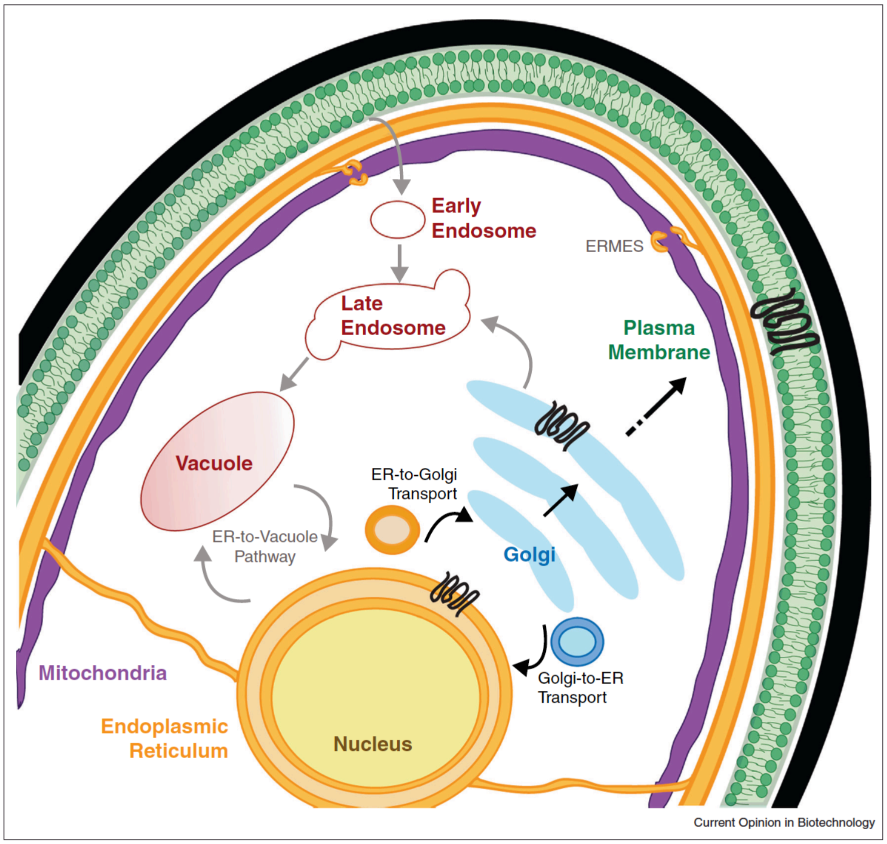


Figure S2 Protein trafficking in yeast. Endoplasmic reticulum (ER) to Golgi to plasma membrane trafficking is shown for membrane proteins. Reprinted with permission from Young and Robinson [1].


1. Young, C. L.; Robinson, A. S., Protein folding and secretion: mechanistic insights advancing recombinant protein production in S. cerevisiae. *Curr Opin Biotechnol* **2014,** 30, 168-77.
